# Docetaxel with or without estramustine for estramustine refractory castration-resistant prostate cancer: a single institution experience

**DOI:** 10.1186/1471-2490-12-3

**Published:** 2012-02-22

**Authors:** Kazuhiko Nakano, Shigeyuki Ohta, Kenji Komatsu, Taro Kubo, Akinori Nukui, Kazumi Suzuki, Shinsuke Kurokawa, Minoru Kobayashi, Tatsuo Morita

**Affiliations:** 1 Department of Urology, Jichi Medical University, Yakushiji 3311-1, Shimotsuke-city, Tochigi 3290498, Japan

## Abstract

**Background:**

The significance of combination of docetaxel (DTX) with estramustine phosphate (EMP) in castration-resistant prostate cancer (CRPC) patients remains unclear. In this study, we aimed to retrospectively evaluate the efficacy and toxicity of DTX with or without EMP and to elucidate the significance of DTX and EMP combination therapy in Japanese EMP-refractory CRPC patients.

**Methods:**

To compare the efficacy and toxicity of DTX and EMP, we divided CRPC patients, who were confirmed to be resistant to EMP, into the following two groups: group D (n = 28), which included patients treated with DTX (60 mg/m^2^, once in every four weeks) alone, and group DE (n = 33), which included patients treated with a combination of DTX (60 mg/m^2^, once in every four weeks) and EMP (twice daily oral administration at 280 mg).

**Results:**

Prostate specific antigen (PSA) response (> 50% decline in PSA) was observed in six patients (21%) in group D and eight patients (24%) in group DE. The median time to progression (TTP) was 12.0 months and 6.2 months and the median overall survival (OS) was 26.4 months and 24.3 months in group D and DE, respectively. There was no statistical difference between the two groups in terms of PSA response, TTP, and OS. The incidence of adverse events of grade 3/4 was low in both the groups, and there was no statistical difference between the two groups.

**Conclusions:**

Although treatment with DTX at 60 mg/m^2 ^was effective and highly tolerated in EMP-refractory Japanese CRPC patients, the DTX and EMP combination therapy might not exhibit any survival benefit for CRPC patients.

## Background

The efficacy of docetaxel (DTX) in castration-resistant prostate cancer (CRPC) patients was shown in two clinical trials of TAX 327 [1] and SWOG 9916 [2] in 2004. Thereafter, DTX-based therapies have been used worldwide for treating CRPC patients. TAX 327 reported a median overall survival (OS) of 18.9 months with administration of DTX every three weeks, and SWOG 9916 reported a median OS of 17.5 months with DTX and estramustine phosphate (EMP) combination therapy every three weeks. While these results showed that DTX was more effective than mitoxantrone, they also raised other questions about whether addition of EMP to DTX was effective for CRPC patients. To clarify this point, several clinical trials and one meta-analysis have been performed thus far. While there have been some reports describing the usefulness of DTX and EMP combination therapy [3-5], other reports suggest that the combination treatment is not useful [6]. A preclinical report showed that addition of EMP did not enhance the efficacy of DTX [7], while other preclinical studies confirmed that EMP in combination with DTX exerted beneficial effects in prostate cancer [8,9]. Since the views on the efficacy of DTX and EMP combination therapy are controversial, it is important to determine clinically whether or not EMP enhances the efficacy of DTX.

In considering the patient backgrounds in the clinical trials described above, however, CRPC patients pretreated with EMP were excluded in SWOG 9916, while both patients treated with and without EMP were enrolled in the clinical trials described above^4-6^. Since patients entered in these previous clinical trials were EMP-naive patients or the mixture of EMP-naive and refractory patients, we focused on the efficacy of DTX by the addition of EMP in EMP-refractory CRPC patients in order to examine the modulatory effect of EMP on DTX in clinical setting.

In the present study, we retrospectively compared the efficacy and toxicity of DTX treatment with and without EMP in Japanese EMP-refractory CRPC patients in order to elucidate the significance of the addition of EMP to DTX therapy.

## Methods

### Subjects

Between July 2003 and October 2010, 90 CRPC patients were pathologically diagnosed with adenocarcinoma of the prostate and treated with DTX at our institution. From this group, 61 CRPC patients who were treated with EMP monotherapy, then confirmed to be resistant to EMP, and received two or more courses of DTX therapy were included in the present analysis. These EMP-refractory CRPC patients were divided into two groups as follows: group D consisting of 28 patients who were treated with DTX without EMP and group DE consisting of 33 patients treated with DTX and EMP combination. The reasons why EMP was not concomitantly administered in group D included adverse events in 16 patients (57%) and the patient request in 12 patients (43%). DTX therapy was initiated in patients resistant to androgen deprivation therapy, which uses luteinizing hormone-releasing hormone (LHRH) analogue and antiandrogen. Patients with antiandrogen withdrawal syndrome were excluded from the study. The inclusion criteria were Eastern Cooperative Oncology Group (ECOG) performance status (PS) of two or lower, white blood cell (WBC) count ≥1500/mm^3^, hemoglobin (Hb) ≥8 g/dL, platelet count ≥100000/mm^3^, total bilirubin ≤ upper limit of normal (ULN), and aspartate aminotransferase (AST) and alanine aminotransferase (ALT) ≤2.5 × ULN.

The patient characteristics by group are shown in Table 1. There was no statistical difference in all parameters between both the groups. The median observation period was 19.5 months (range; 3.0-60.9 months). The median number of DTX treatment course was four in group D and five in group DE. This study was approved by the institutional review board of Jichi Medical University and written informed consent was obtained from all patients.

**Table 1:** Patients' characteristics

	Docetaxel with estramustine (n = 33)	Docetaxel without estramustine (n = 28)	p value*
Age (years), median (range)	67 (55-88)	72 (50-82)	0.062
PSA at PCa diagnosis (ng/ml), median(range)	124.1 (10.3-4116.0)	102.1 (4.7-19523.1)	0.452
PSA at baseline (ng/ml), median(range)	17.1 (0.6-1053.0)	11.4 (0.8-618.4)	0.633
Time from diagnosis to this chemothrapy (months), median(range)	34 (4-176)	37 (7-113)	0.492
ECOG performance status, n (%)			0.299
0	19 (58)	12 (43)	
1	9 (27)	13 (46)	
2	5 (15)	3 (11)	
Gleason score, n (%)			0.517
< 6	2 (6)	2 (7)	
7	9 (27)	4 (14)	
> 8	20 (61)	19 (68)	
unknown	2 (6)	3 (11)	
Metastatic site, n (%)			
Bone	17 (52)	16 (57)	0.797
Lymph nodes	10 (30)	11 (39)	0.590
Liver	0 (0)	1 (4)	0.459
Lung	3 (9)	0 (0)	0.243
None	12 (36)	7 (25)	0.080
Prior treatment, n (%)			
MAB	33 (100)	28 (100)	ne
Estramustine	33 (100)	28 (100)	ne
Radical Prostatectomy	2 (6)	1 (4)	1.000
Radiation Therapy	4 (12)	3 (11)	1.000
Dexamethasone	8 (24)	11 (39)	0.270
Other Chemotherapy	6 (18)	8 (29)	0.375
No. of cycles, median (range)	5 (2-32)	4 (2-27)	0.382

* by Mann-Whitney *U *test and chi-square test, PSA, prostate-specific antigen; PCa, Prostate Cancer; CRPC, Castration Resistant Prostate Cancer; ECOG, Eastern Cooperative Oncology Group; MAB, Maximum Androgen Blockage; ne, not evaluable

### Treatment

We used a modified version of the regimen used in SWOG 9916 [2] as the treatment protocol. Briefly, DTX 60 mg/m^2 ^was administered by intravenous drip infusion for one hour on day one once every four weeks. Further, eight mg of dexamethasone was administered by intravenous drip infusion as premedication before and after treatment with DTX. The treatment course was repeated every four weeks. In group D, DTX alone was administered after confirming the resistance to EMP. Meanwhile, in group DE, DTX and EMP equivalent to the amount administered before DTX therapy was administered after confirming the resistance to EMP. Although 280 mg EMP twice daily was orally administered every day, dose reduction up to 280 mg/day was allowed according to the degree of adverse events. The treatment was continued until progression of disease was observed, and it was stopped after the occurrence of adverse events which made it impossible to continue the treatment or on patient request. Progression of disease was defined as biological progression characterized by the increase in evaluable lesions observed on imaging test or continuous elevation of serum PSA on at least three consecutive measurements.

At the initiation of treatment and before each cycle, medical history of the patient was taken and physical examination, PS evaluation, and hematological examination (blood count, biochemistry, and PSA) were performed at least once every four weeks. Computed tomography (CT) and bone scintigraphy were performed at least once every 16 weeks.

### Outcomes

Primary endpoint was PSA response. According to the recommendations of Prostate Cancer Clinical Trial Working Group [10], the decreasing rate of PSA of each patient is shown in the waterfall plot. The decreasing rate of PSA was obtained from the value determined just before the initiation of treatment with DTX and the lowest value of PSA during treatment. Patients with a PSA decrease rate of 50% or more were defined as PSA responders and those with a rate of 0% or less were PSA nonresponders.

Secondary endpoints were time to progression (TTP) of PSA, OS, and toxicity. TTP was defined as the period from the initiation of treatment to PSA progression. PSA progression was defined as a 25% or greater increase and an absolute increase of 2 ng/mL or more from the nadir, which was confirmed by a second value obtained three or more weeks later. OS was defined as the period from the initiation of treatment to death. When patients were lost to follow-up, OS was considered up to the last day on which the patient survival was confirmed.

Adverse events were determined according to the classification by National Cancer Institute Common Toxicity Criteria (NCI-CTC), version 4.

### Statistics

Mann-Whitney *U *test or chi-square test was used for comparing clinical and pathological data, PSA response, and adverse events. TTP and OS were obtained by using Kaplan-Meier method and compared using log-rank test. Multivariate analysis was performed by using Cox proportional-hazard analysis for the identification of prognostic factors. Continuous data were divided into two groups according to median value. P value of less than 0.05 was judged as statistically significant.

## Results

### Efficacy

**Figure 1:**
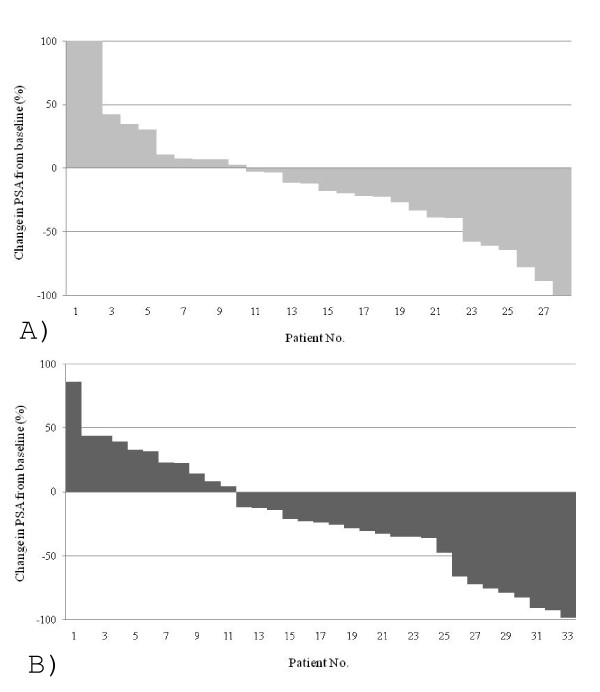
**Waterfall plots of% change in PSA after treatment by docetaxel without estramustine (n = 28; A) and with estramustine (n = 33; B)**.

PSA response by group is shown in a waterfall plot (Figure 1). Six patients (21%) in group D and eight patients (24%) in group DE were PSA responders and 10 patients (36%) in group D and 11 patients (33%) in group DE were PSA nonresponders. There was no statistical difference in PSA response between these two groups (p = 0.962).

**Figure 2:**
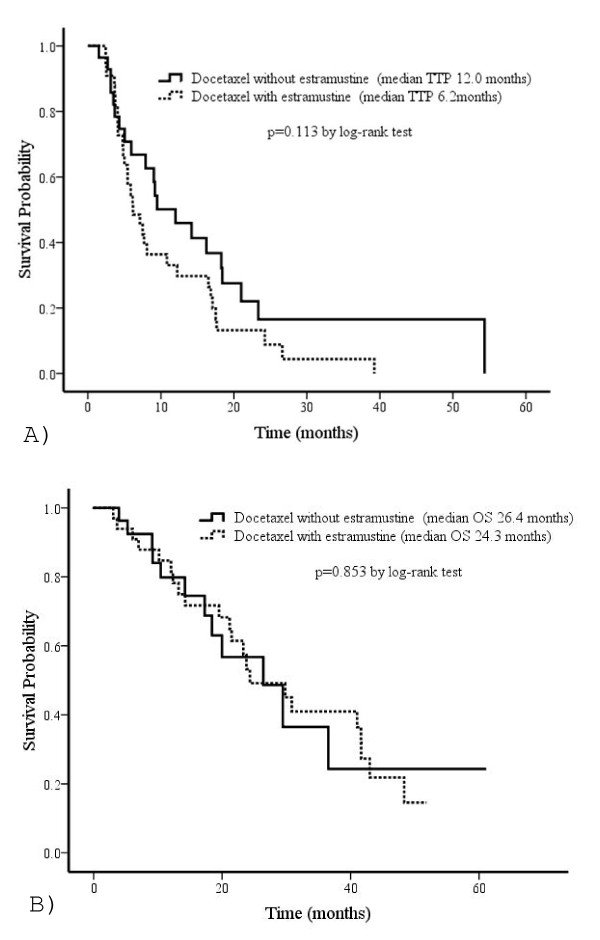
**Time to PSA progression (A) and overall survival (B) in patients treated by docetaxel with or without estramustine**.

The median TTP was 12.0 months (range; 1.5-54.3 months) in group D and 6.2 months (range; 2.4-39.2 months) in group DE (Figure 2A); further, the median OS was 26.4 months (range; 3.9-60.9 months) in group D and 24.3 months (range; 3.0-51.8 months) in group DE (Figure 2B). There were no statistical differences in median TTP and OS between these two groups (TTP, p = 0.113; OS, p = 0.853). Eight patients (29%) in group D and 19 patients (58%) in group DE died of cancer. The reasons for discontinuation were progression of disease in 18 patients (64%) in group D and 22 patients (67%) in group DE and the occurrence of adverse events in three patients (11%) in group D and one patient (3%) in group DE. Currently, four patients (14%) in group D and six patients (18%) in group DE are continuing the treatment.

### Subgroup analysis

Univariate analysis using log-rank test revealed a significant association between OS and five factors (PSA at baseline, ECOG performance status, time from diagnosis to DTX chemotherapy, bone metastasis, and the number of DTX cycles). These five factors were used in multivariate analysis. A PSA value > 12.8 ng/mL at baseline indicated significantly worse prognosis, and 35 months or more period from the diagnosis to DTX chemotherapy and five or more cycles of DTX suggested significantly better prognosis. The presence or absence of EMP with DTX was not a prognostic factor (Table 2). Multivariate analysis using six factors, which included the five above-mentioned factors and treatment factor (group D vs group DE), revealed the same results as shown in Table 2, indicating that addition of EMP to DTX is not associated with prognosis (data not shown).

**Table 2:** Prognostic factors for overall survival by univariate and multivariate Cox regression analysis

			Univariate				Multivariate			
parameter	category	n	Hazard ratio	95%CI		p value	Hazard ratio	95%CI		p value
Treatment	D	28	1.000			0.853				
	DE	33	0.934	0.454	1.923					
Age (years)	≦70	30	1.000			0.549				
	> 70	31	0.808	0.401	1.628					
Gleason Score	≦7	17	1.000			0.259				
	> 8	39	1.558	0.718	3.381					
PSA at PCa diagnosis (ng/ml)	≦113	31	1.000			0.940				
	> 113	30	1.027	0.520	2.025					
PSA at baseline (ng/ml)	≦12.8	31	1.000			< 0.001	1.000			0.017
	> 12.8	30	4.903	2.181	11.024		3.605	1.262	10.296	
ECOG performance status	≦1	53	1.000			< 0.001	1.000			0.218
	2	8	5.567	2.366	13.098		1.803	0.706	4.605	
Time from diagnosis to this chemothrapy (months)	≦35	31	1.000			0.003	1.000			0.015
	> 35	30	0.351	0.172	0.715		0.286	0.104	0.782	
Bone metastasis	no	28	1.000			0.014	1.000			0.419
	yes	33	2.383	1.165	4.873		0.646	0.224	1.862	
No. of Docetaxel cycles	< 5	28	1.000			< 0.001	1.000			< 0.001
	<5	33	0.213	0.100	0.452		0.209	0.093	0.472	
Hemoglobin (g/dl)	≦12.0	33	1.000			0.120				
	> 12.0	28	0.580	0.289	1.163					

CI, confidence interval; D, Docetaxel without estramustine; DE, Docetaxel with estramustine; PSA, prostate-specific antigen; PCa, Prostate Cancer; ECOG, Eastern Cooperative Oncology Group; CRPC, Castration Resistant Prostate Cancer

### Toxicity

Adverse events are shown in Table 3. There was no statistical difference between the two groups based on the incidence of grade 3/4 adverse events. The most frequent adverse events were neutropenia (n = 19, 68%) in group D and leukopenia (n = 15, 45%) in group DE. The most frequent grade 3/4 adverse event was neutropenia in the both groups; 14 patients (50%) in group D and 11 patients (33%) in group DE had neutropenia. Although there were no significant differences in the incidence of leukopenia and neutropenia between the two groups, these effects were less common in group DE than in group D. Further, grade 2 thromboembolism in one patient and interstitial pneumonia in one patient were observed in group DE; however, there was no treatment-related death in any group. In addition, gastrointestinal symptoms (anorexia, nausea, and diarrhea), fatigue, and alopecia were frequently observed in the both the groups. Grade 3 allergic reaction was observed in one patient in group DE and taste disorder was observed in two patients (one patient in group D and one patient in group DE).

**Table 3:** Acute adverse events according to NCI-CTC

	Docetaxel with estramustine				Docetaxel without estramustine				
	(n = 33)				(n = 28)				
		
	Any grade*		Grade3-4		Any grade		Grade3-4		p value**
		
	n	%	n	%	n	%	n	%	
Leukopenia	15	45	7	21	18	64	11	39	0.123
Neutropenia	13	39	11	33	19	68	14	50	0.187
Febrile neutropenia	0	0	1	3	0	0	0	0	0.353
Anemia	9	27	1	3	5	18	2	7	0.443
Thrombocytopenia	0	0	0	0	0	0	0	0	ne
Fatigue	10	30	0	0	10	36	0	0	ne
Anorexia	10	30	0	0	10	36	1	4	0.274
Nausea	13	39	2	6	10	36	0	0	0.185
Diarrhea	4	12	0	0	2	7	0	0	ne
Taste alteration	1	3	0	0	1	4	0	0	ne
Dizziness	3	9	0	0	2	7	0	0	ne
Flushing	2	6	0	0	0	0	0	0	ne
Edema	1	3	0	0	2	7	0	0	ne
AST/ALT	1	3	0	0	0	0	0	0	ne
Allergic reaction	1	3	1	3	1	4	0	0	0.353
Pulmonary fibrosis	1	3	1	3	0	0	0	0	0.353
Hair loss	14	42	0	0	12	43	0	0	ne
Mood alteration	1	3	0	0	0	0	0	0	ne
Neuropathy	1	3	0	0	1	4	0	0	ne
Thromboembolic event	1	3	0	0	0	0	0	0	ne

* Grade by National Cancer Institute-Common Toxicity Criteria; **indicates the difference of the rate of grade 3-4 adverse events between two regimens by chi-square test; ne, not evaluable

## Discussion

The significance of addition of EMP to DTX therapy in CRPC patients is still controversial in clinical trials [3-6] as well as preclinical studies [7-9]. Therefore, it is reasonable to compare the clinical efficacy of DTX + EMP combination with DTX alone in EMP-refractory CRPC patients in order to elucidate the significance of addition of EMP to DTX therapy. Present retrospective study showed, for the first time, that there were no significant differences in OS, TTP, PSA response, and adverse events between group D and group DE in EMP-refractory CRPC patients, suggesting that addition of EMP to DTX therapy had no survival benefit.

The PSA response (> 50% decline in PSA) in SWOG 9916 [2] was 50%. However, the PSA response in this study was low; only 21% patients in group D and 24% patients in group DE responded to treatment with 60 mg/m^2 ^DTX, which was similar to the regimen of SWOG 9916. Our result is consistent with that of another study in which patients who had been treated with EMP showed a lower PSA response as compared to patients without a history of EMP treatment [11], suggesting that treatment with EMP before DTX treatment decreases the PSA response. However, the median OS in group D was 26 months and that in group DE was 24 months. Further, these median OS are not only greater than that of nine months after treatment with 55 mg/m^2 ^DTX [12] or that of 18 months after treatment with 60 mg/m^2 ^DTX in SWOG 9916 but also consistent with the 15-27 months median OS obtained after treatment with 70-75 mg/m^2 ^DTX [1,4-6,13,14]. The median OS of 24 months or more achieved in this study was considered to be due to the initiation of DTX therapy with low PSA value as compared with that obtained in the previous reports [1,4-6,13,14], because multivariate analysis performed in this study and the report by Bamias et al. [15] demonstrated that OS prolonged when PSA was low at the initiation of DTX therapy. Since the adverse events observed in this study were well tolerated, treatment with 60 mg/m^2 ^DTX seems to be effective and safe even in EMP-refractory CRPC patients. In recent years, several novel agents such as abiraterone [16], sipuleucel-T [17] and MDV3100 [18] have been developed and showed significant clinical efficacy against CRPC. Treatment with these agents would provide the survival benefit for CRPC patients.

Regarding adverse events in TAX 327 and SWOG 9916, the incidences of grade 3/4 neutropenia were 32% and 13% in TAX 327 [1] (DTX, 75 mg/m^2^) and SWOG 9916 [2] (DTX, 60 mg/m^2^), respectively. Further, the incidence of grade 3/4 neutropenia in clinical trials performed in Japan was 93% and 33% after treatment with 70 mg/m^2 ^[19] and 55 mg/m^2 ^DTX [12], respectively, indicating high incidences of DTX toxicity. In addition, the incidence of neutropenia after 60 mg/m^2 ^DTX treatment was 86% in a clinical study on Japanese pancreatic cancer patients [20], suggesting that the incidence of neutropenia is high among Japanese patients. Although the sample size of our study was small, the incidence of grade 3/4 neutropenia was 50% and 33% in group D and DE, respectively, which were higher as compared to the values reported in SWOG 9916 using 60 mg/m^2 ^DTX. However, no incidence of death or severe complications suggested that our treatment was well tolerated. Furthermore, grade 2 thromboembolism was observed in one patient (3%) of group DE. Since EMP increases the incidence of thromboembolism [2,3,6], it should be used with caution. The reason why the incidence of adverse events was not significant between group D and DE might be due to the means of treatment assignment. Namely, two-thirds of patients with adverse events by EMP monotherapy and those without them were classified in group D and DE, respectively. Although addition of EMP to DTX therapy might not show any survival benefit in CRPC patients, it was of interest that addition of EMP to DTX therapy lowered the incidence of grade 3/4 neutropenia (50% in group D and 33% in group DE), though not by a significant extent. Meta-analysis [3] of chemotherapy combined with EMP showed a significantly low incidence of neutropenia due to concomitant use of EMP in the chemothrapy. In addition, treatment with EMP showed increase in the leukocyte count in patients with hormone-naïve prostate cancer [21] or CRPC [22]. Taken together, it is suggested that EMP increases the leukocyte count leading to the myeloprotection in the chemotherapy, albeit under limited conditions; hence, the significance of addition of EMP to DTX therapy may be attributed to the myeloprotection rather than the survival benefit. Further studies are required to clarify this point.

Multivariate analysis performed in this study revealed that PSA at baseline, time from diagnosis to DTX chemotherapy and the number of DTX cycles were related to the prognosis. Although these factors were statistically significant, the definitive conclusions cannot be drawn because of the small number of death events observed in this study. These factors have been reported as prognostic factors in other studies [11,15,23,24]; hence, DTX treatment should be introduced when PSA values are low and continued for as long as possible. However, addition of EMP did not serve as a prognostic factor.

This study has some limitations; it is a retrospective study with a small sample size and not a randomized clinical trial. However, it was concluded that addition of EMP to DTX therapy might not show any survival benefit in EMP-refractory CRPC patients although treatment with 60 mg/m^2 ^DTX had the OS and toxicity profiles comparable to those in TAX 327 and SWOG 9916. Further studies would be needed to elucidate the significance of addition of EMP to DTX therapy.

## Conclusions

Although treatment with DTX at 60 mg/m^2 ^was effective and highly tolerated in EMP-refractory Japanese CRPC patients, addition of EMP to DTX therapy might not show any survival benefit in CRPC patients.

## Competing interests

The authors declare that they have no competing interests.

## Authors' contributions

This study has been designed by KN and TM. The clinical database of the patients have been acquired by KN, SO, KK, TK, AN, KS, SK, MK and TM. Manuscript has been written by KN and TM. KN is responsible for the statistical analyses. Conclusions have been drawn mainly by KN and TM. TM has given final approval of the version to be published. All authors read and approved the final manuscript.

## Pre-publication history

The pre-publication history for this paper can be accessed here:

http://www.biomedcentral.com/1471-2490/12/3/prepub
